# New lung mass in a patient with granulomatosis with polyangiitis

**DOI:** 10.1007/s00296-020-04646-w

**Published:** 2020-07-16

**Authors:** Anna Masiak, Jadwiga Fijałkowska, Szymon Nowakowski, Żaneta Smoleńska, Zbigniew Zdrojewski

**Affiliations:** 1 Department of Internal Medicine, Connective Tissue Diseases and Geriatrics, Medical University of Gdansk, ul. Dębinki 7, 80-952 Gdansk, Poland; 2 Second Department of Radiology, Medical University of Gdansk, Gdansk, Poland

**Keywords:** Granulomatosis with polyangiitis, Lung neoplasm, Carcinogenesis

## Abstract

Granulomatosis with polyangiitis (GPA) is a potentially lethal ANCA-associated small-vessel vasculitis characterized by a typical triad of upper respiratory tract, lung, and kidney involvement. Lung involvement in GPA occurs in 25–80% of cases. The most common radiographic and computed tomography (CT) abnormalities of pulmonary GPA are lung nodules and masses, very often multiple and with cavitation. As there are various clinical presentations, the diagnosis of GPA can be challenging, and the illness is difficult to distinguish from other diseases such as infection or malignancy. Following the improved survival rates in patients with GPA, there is accumulating evidence to suggest an increased occurrence of different types of cancer. Exposure to cyclophosphamide seems to be one of its main causes. We present the case of a patient with chronic GPA who was hospitalized owing to a new infiltrate in the lung, suggesting relapse of the disease, and finally diagnosed with small cell lung cancer. Data regarding lung cancer in GPA patients are limited. While there are some case reports and short case series in the literature, there are no detailed data regarding an association between CYC exposure and lung cancer development in vasculitis. It is necessary to consider the causes of pulmonary masses other than a GPA relapse. Bronchoscopy with biopsy and histopathological examination are crucial in proper differential diagnosis. GPA patients require long-term follow-up to monitor for the development of complications during treatment.

## Introduction

Granulomatosis with polyangiitis (GPA) is a rare, potentially lethal, multisystem disease that belongs to the group of primary systemic ANCA-associated small-vessel vasculitides (AAV). Granulomatous inflammation and necrotizing vasculitis of small blood vessels lead to diverse clinical presentations with a classic triad of symptoms involving the upper and, lower respiratory tract as well as the kidneys. The disease can occur in several forms, from mild to very severe, and can be life threatening. With a standard therapy regimen, remission can be induced in about 70–90% of patients, but the typical course of the disease is with remissions and exacerbations, which lead to the necessity for repeated courses of immunosuppressive treatment [1–4]. Pulmonary involvement in GPA occurs in 25–80% of cases [5]. The most common radiographic and computed tomography (CT) abnormalities of pulmonary GPA are lung nodules and masses, very often multiple and with cavitation. Infiltrates, air-space, and ground-glass opacities are also frequent findings. This variety in clinical presentation can make the diagnosis of GPA challenging and it can be difficult to distinguish it from other diseases such as an infection, sarcoidosis, or malignancy [6, 7].

We present a case of a patient with chronic GPA, who was hospitalized as a result of new infiltrate in the lung suggestive of a relapse of the disease, and who was finally diagnosed with small cell lung cancer. Following this case report, we discuss the risk of the development of lung cancer in GPA.

The patient has provided informed consent for publication of the case.

## Methods

### Search strategy

**Fig. 1:**
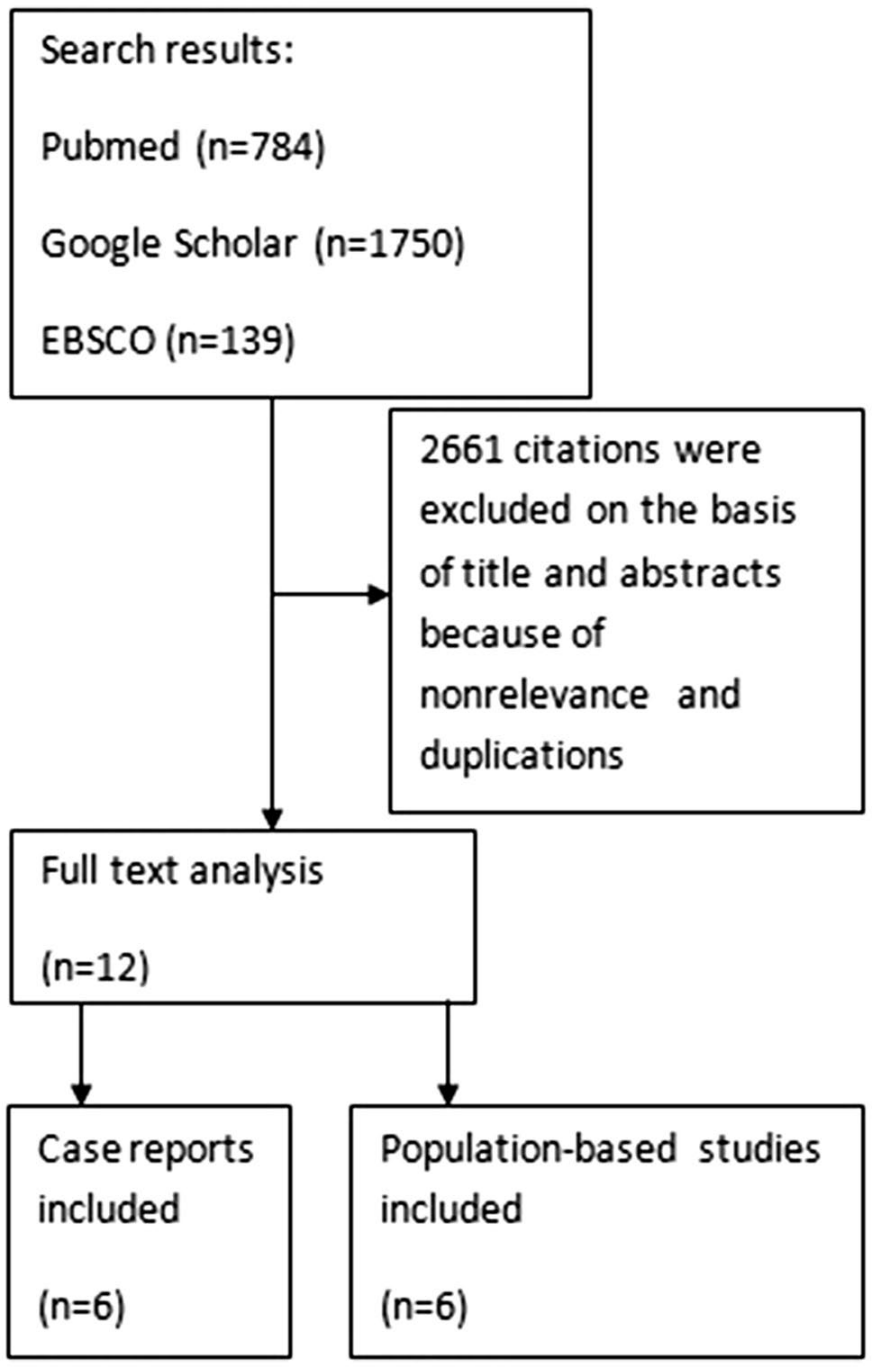
Search strategy

A literature search for patients with GPA and lung cancer was carried out using MEDLINE/PubMed, Google Scholar, and EBSCO, with no time limit. The search was conducted using the following keywords: “granulomatosis with polyangiitis”, “lung neoplasm,” and “carcinogenesis” (Fig. 1). Using a combination of these search terms, we undertook a systematic review of the literature published in English, limited to full-text publications of original articles, letters to the editor, and case reports in peer-reviewed journals, for a discussion and analysis of studies reporting lung cancer development in GPA. We identified six case reports and summarize the findings in Table 1. Lung cancer prevalence in patients with ANCA-associated vasculitis is presented in Table 2.

**Table 1:** Review of granulomatosis with polyangiitis cases associated with lung cancer

Year, author	No. of cases with lung cancer	Age (years)/gender	Organ involvement in GPA	Time interval between GPA diagnosis and detection of cancer (years)	Cumulative cyclophosphamide dose (g)	Smoking	Outcome
Campainha (2013) [3]	1	45, M	Lung	GPA 15 months after cancer treatment	NR	Yes	Died
López (2008) [18]	1	50, M	Kidney	23	NR	NR	NR
Toriyama (2018) [19]	1	65, M	Lung	18	NR	NR	NR
Doberstein (2017) [20]	1	69, M	Eye, lung	2	NR	Yes	Died
Xie (2019) [21]	1	78, M	Lung, kidney	1	NR	Yes	NR
Yamada (2019) [22]	1	79, M	Lung, kidney, upper respiratory tract	3	NR	NR	Alive

*NR* not reported

**Table 2:** Prevalence of lung cancer among patients with ANCA-associated vasculitis

Year, author	Type of the disease	No. of study group	No. of cases with lung cancer	SIR of lung cancer	95% CI
Knight (2002) [6]	GPA	1065	8	2.0	0.9–3.9
Faurschou (2015) [8]	GPA	293	5	1.1	0.3, 2.5
Rahmattulla (2015) [3]	GPA and MPA	138	2	0.75	0.23–3.30
Życińska (2013) [12]	GPA	117	2	1.7	0.5–3.4
Heijl (2011) [17]	GPA	535	5	1.3	0.4–3.0
Sriskandarajah (2017) [23]	ANCA-associated glomerulonephritis	419	7	1.73	0.83–3.63

*SIR* standardized incidence ratios

## Case report

**Fig. 2:**
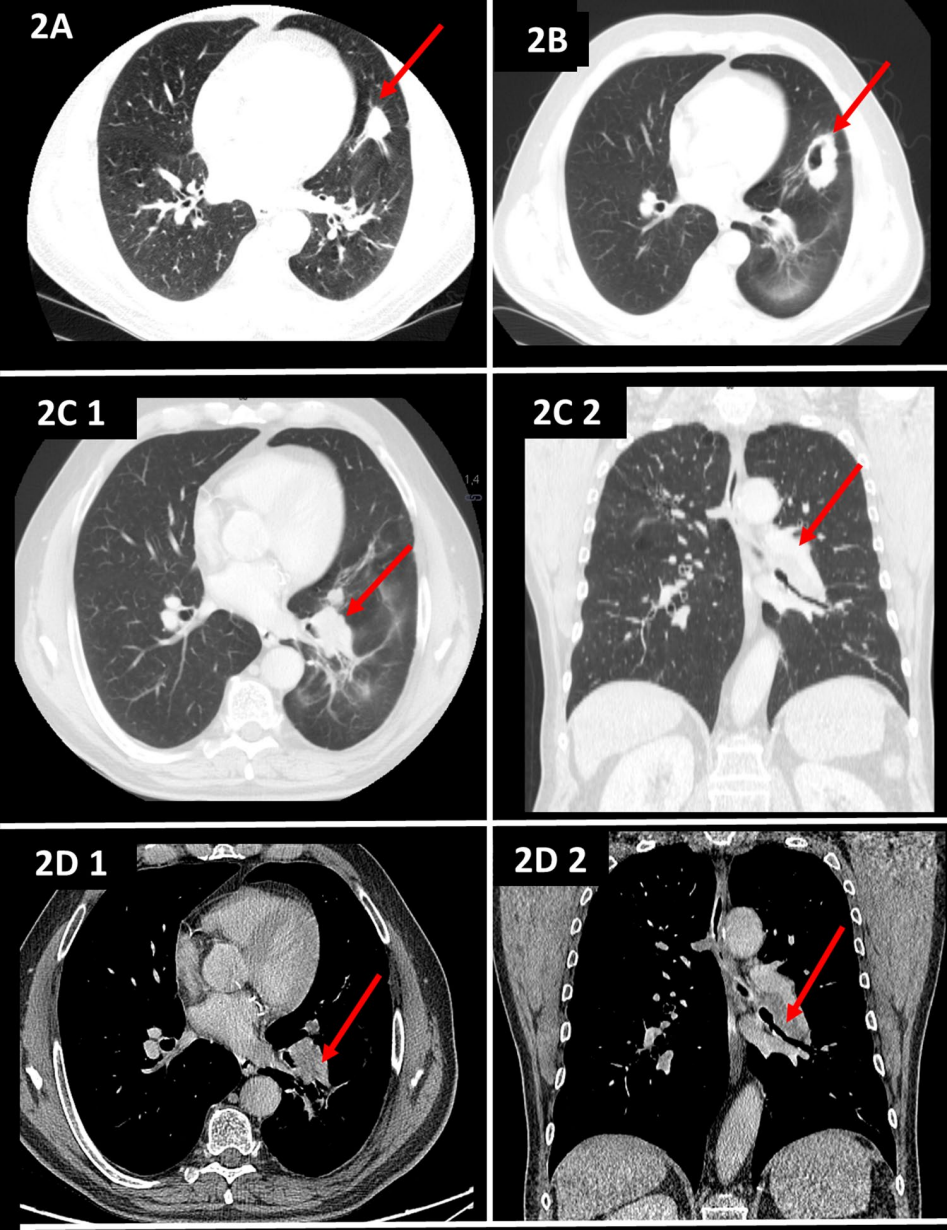
**a** High-resolution CT image revealing a large nodule in the lower lobes. **b** High-resolution CT image revealing a cavitated nodule. **c** CT pulmonary window revealing enlarged lymph nodes in the hilus of the left lung that impress the bronchi to the lower lobe, which, combined with the presence of subpleural nodules, aroused suspicion of a proliferative process (2C1 axial scan, 2C2 coronal scan). **d** CT soft tissue window revealing a pathological mass in the hilus of the left lung (2D1 axial scan, 2D2 coronal scan)

A 63-year-old man had been diagnosed with granulomatosis with polyangiitis (GPA) at the age of 51 on the basis of the presence of a pulmonary nodular mass (Fig. 2a), pansinusitis and cytoplasmic anti-neutrophil cytoplasmic antibody (PR3-ANCA). During the course of the disease, relapses were observed with progression and cavitation of the infiltrates in the lungs (Fig. 2b), exacerbations in the sinus lesions, and the occurrence of an inflammatory orbital pseudotumor. The patient had been treated with prednisone in varying doses, cyclophosphamide (CYC, total dose 70 g orally and 5 g intravenously) and then azathioprine and methotrexate following the diagnosis. Despite the treatment, a persistent orbital tumor, joint pain and exacerbations in the paranasal sinusitis were observed each time steroids were reduced below 10 mg/day. However, there were no new infiltrations in his lungs and PR3-ANCA titers were low (PR3-ANCA 18 RU/ml, *n *< 20 RU/ml). In November 2019, the patient was hospitalized because of headaches, general weakness, decrease in exercise capacity, and a tear of the left eye over 2 months. He did not present fever, hemoptysis or weight loss. Physical examination revealed exophthalmos (stable compared to previous months) and there were no obvious abnormalities during lung auscultation. Laboratory findings on admission showed mild anemia (Hb 12.7 g/dl), slightly elevated inflammatory markers (CRP 7.8 mg/l, *n* < 5 mg/l), and a greatly increased level of PR3-ANCA (PR3 > 200 RU/ml, *n* < 20 RU/ml). A relapse of GPA was suspected and a chest CT scan was performed, which revealed enlarged lymph nodes in the hilus of the left lung that impressed the bronchi to the lower lobe, which, combined with the presence of subpleural nodules, raised concerns regarding proliferation (Fig. 2c, d). Because the patient had a history of smoking 27 packets of cigarettes a year it was decided to perform bronchoscopy. This showed left lower lobe bronchial stenosis with granulomatous hypertrophy of the mucosa. Inflammatory cells, including macrophages, partial hemosiderophages and epithelial cells without atypical features, were found in the sediment from bronchoalveolar lavage. Unexpectedly, histopathology of the tissue that has been narrowing the bronchus revealed the presence of small cell lung cancer. Chemotherapy and radiotherapy were started.

## Discussion

Since the late 1970s, the introduction of glucocorticoids and cyclophosphamide (CYC) as standard therapy in GPA has substantially improved the prognosis, thus increasing cumulative survival at 1, 2, and 5 years to 88%, 85%, and 78%, respectively [8]. However, the disease runs with remissions and exacerbations, resulting in the necessity of repeated courses of immunosuppressive treatment [3]. Alongside the improved survival rates in AAV patients, many reports suggest an increased occurrence of various malignancies [9, 10]. The increased risk of acute myeloid leukemia and bladder cancer is mainly attributed to CYC exposure and its carcinogenic effects and the toxic metabolite acrolein that becomes highly concentrated in the urine [9, 10]. The development of malignancies following CYC use is related to the duration of exposure and the cumulative doses of CYC. A high prevalence of cancer has been demonstrated in AAV patients who received cumulative CYC doses exceeding 36 g or who were treated for more than a year [11–16]. In most of the population-based studies, a pronounced increase was reported in bladder cancer, squamous cell skin cancer, and malignant lymphomas in particular [10, 12, 15–17]. Rahmattulla et al. found in their cohort of 138 patients with AAV a total of 85 malignancies in 36 individuals during a mean follow-up of 9.7 years, resulting in a 2.21-fold higher malignancy risk in comparison with the general population. They found the highest risk for non-melanoma skin cancers (NMSC), with a standardized incidence ratio of 4.23, but the incidence rates of other malignancies were not significantly increased [16]. Meta-analysis performed by Shang et al. shows that patients treated with CYC were at increased risk of late-occurring malignancies, particularly of NMSC, leukemia and bladder cancer, but not of kidney, prostate, colon and breast cancers [18]. Data from several studies suggest a standardized incidence ratio of cancer in AAV from 1.6 to 3.8 compared to that of the general population [13, 19]. An important measure in reducing cancer risk in patients with AAV has been the replacement of CYC with azathioprine for maintenance treatment. This change in practice occurred after the CYCAZAREM study in 2003 [20]. Recent studies suggest that with a reduction in CYC dosage, only NMSC risk remains increased [21].

Data regarding lung cancer in GPA patients are limited. There are some case reports and short case series in the literature [7, 22–26]. López et al. presented a patient with ANCA-positive GPA, who developed lung cancer that imitated GPA relapse. The ANCA were negative at the time of cancer diagnosis, suggesting immunological remission of vasculitis [22]. Toriyama et al. reported an interesting case of GPA with lung cancer that developed while taking long-term cyclophosphamide. The authors did not refer to ANCA status during the cancer diagnosis [23]. Our patient had a definite increase in ANCA titer which, with the presence of new infiltrations in the lungs, strongly suggested GPA relapse. Differential diagnosis of lung mass or cavitary lung disease is extensive and includes, for instance, various infections, autoimmune conditions, and primary and metastatic malignancies [27]. GPA, although rare, can also be a paraneoplastic syndrome [28]. In many published cases, it was only the prolonged detailed diagnostics, based on imaging and histopathological examinations, that made it possible to determine the proper diagnosis and appropriate treatment [29–31]. It remains currently unknown whether there is an association between solid tumors and elevated serum levels of ANCAs [32, 33].

The link between lung cancer and GPA has not yet been investigated. The scale of the problem is illustrated by data from population-based studies. Sriskandarajah et al. found, on the basis of data from 419 patients with ANCA-associated glomerulonephritis, 46 cancer cases in 41 (9.5%) patients with 7 lung cancers [34]. A study in Denmark linking the identification of cancer in 293 granulomatosis with polyangiitis patients from 1973 to 1999 who were followed through to 2010, showed 73 cancers with 5 lung cancers among them (SIR 1,1) [12]. Jardel et al. in a retrospective analysis of the French Vasculitis Study Group registry found that lung cancer was the most common cause of death due to malignancy in systemic necrotizing vasculitides [35]. Many chronic primary autoimmune diseases have been associated with an increased risk of de novo cancer development [36–39]. There was found to be a fourfold risk of lung cancer in patients from Sweden with systemic sclerosis, discoid lupus erythematosus, and polymyositis/dermatomyositis [40]. Yu et al. found an increased prevalence of lung cancer in patients with systemic sclerosis, Sjögren syndrome, lupus, and dermatomyositis, particularly in those with pulmonary involvement during the disease [41].

Detailed studies of lung cancer formation in vasculitis have not, to our knowledge, been carried out. Several potential mechanisms of increased malignancy risk in vasculitis have been suggested [42]. Evidence for CYC exposure as a lung carcinogen remains inconclusive. Cyclophosphamide is one of the immunosuppressive agents used as part of a chemotherapy regimen in lung cancer [43]. Pulmonary side effects of CYC are rare (< 1%) and are manifest either as an early-onset pneumonitis or as a late fibrosis [44]. There are no data regarding lung mass or lymphadenopathy induction by CYC.

The immune system dysfunction associated with autoimmunity may increase the risk of certain cancers [45, 46]. Dysregulation of both the innate and the adaptive immune system might have led to the observed associations between autoimmunity and cancer. Chronic inflammation promotes genetic and epigenetic aberrations, with various pathogeneses, and the actual mechanism of the autoimmune or inflammatory disease, such as the type of cells mediating the inflammation, seems not to affect cancer risk significantly [47]. An exaggerated anti-self-tissue immune response appears to cause the damage with subsequent inflammation leading to focal and systemic malignancy [46]. Pathogenic mechanisms with continuous accumulation and the proliferation of differentiated fibroblasts in the regions of repeated epithelial injury, connected with decreased apoptosis as well as pulmonary fibrosis, seem very similar to those followed by cancer cells, featuring unrestricted cell multiplication, immortality, or rapid immigration [41]. Uncontrolled inflammation can become chronic, prompting cellular events that induce malignant cell transformation and carcinogenesis in surrounding tissues. The observation that tumors generally arise in the inflammatory tissue underlines the importance of the role of the local inflammatory mediators in carcinogenesis [41]. In AAV, inflammation at disease sites is perpetuated by necrosis of the blood vessel walls and infiltration of immune cells into damaged organs. Immune aberrations in T cell response, in terms of both the cytokine profile (Th1/Th2/Th17) and of Tregs, play a major role in the pathogenesis of GPA [48]. Interleukin (IL)-17 and IL-23 play roles in inflammation and autoimmunity and are increased in patients with ANCA-associated vasculitis [49]. Recent studies have shown that the serum IL-23 level, but not that of IL-17, is also elevated in small cell lung cancer [50, 51]. As lung cancer is not a common event in GPA patients, it seems that there might be another possible triggering factor.

Established environmental risk factors for lung cancer include smoking cigarettes and other tobacco products, occupational lung carcinogens, radiation and air pollution. Smoking is one of the environmental factors that play an important role in the genesis of aberrant immune response and the development of different inflammatory diseases and has immuno-modulatory effects in several chronic inflammatory disorders [52]. Smoking is associated with a more intensive development of inflammatory diseases and is a significant and dose-dependent risk factor for relapse. Above all, cigarette smoking is the predominant cause of lung cancer and the leading worldwide cause of cancer death [52–57].

In the patient whose case has been presented, the cumulative dose of CYC was about 75 g, which, in combination with a smoking habit, a family history of lung cancer (the patient’s sister), repeated exposure to X-rays (several lung and head-CTs were carried out during the course of GPA), could be associated with cancer development. It cannot, of course, be ruled out that the cancer as an accompanying disease was accidental in character.

Despite symptoms suggesting an exacerbation of vasculitis, such as a new mass and a high level of ANCA, it is still necessary to consider causes of pulmonary masses other than GPA relapse. It is also necessary to further evaluate the drugs used to treat GPA and their impact on the mechanisms leading to carcinogenesis. There is a continued need for alternatives to CYC that are less toxic and for cancer screening in patients with a high risk of neoplasm development [58]. Bronchoscopy with biopsy and histopathological examination are crucial in proper differential diagnosis. GPA patients require long-term follow-up to monitor the possible development of complications during the treatment.

## References

[1] Cortazar FB, Muhsin SA, Pendergraft WF (2018). Combination therapy with rituximab and cyclophosphamide for remission induction in ANCA vasculitis. Kidney Int Rep.

[2] Jones RB, Tervaert JWC, Hauser T (2010). Rituximab versus cyclophosphamide in ANCA-associated renal vasculitis. N Engl J Med.

[3] Walsh M, Flossmann O, Berden A (2012). Risk factors for relapse of antineutrophil cytoplasmic antibody-associated vasculitis. Arthritis Rheum.

[4] Miloslavsky EM, Specks U, Merkel PA (2015). Outcomes of nonsevere relapses in antineutrophil cytoplasmic antibody-associated vasculitis treated with glucocorticoids. Arthritis Rheumatol (Hoboken, NJ).

[5] Mohammad AJ, Mortensen KH, Babar J (2017). Pulmonary involvement in antineutrophil cytoplasmic antibodies (ANCA)-associated vasculitis: the influence of ANCA subtype. J Rheumatol.

[6] Masiak A, Struk-Panfill M, Zdrojewski Z (2015). Infectious complication or exacerbation of granulomatosis with polyangiitis?. Reumatologia.

[7] Campainha S, Gonçalves M, Tavares V (2013). Granulomatose com poliangeíte inicialmente diagnosticada como cancro do pulmão. Rev Port Pneumol.

[8] Flossmann O, Berden A, de Groot K (2011). Long-term patient survival in ANCA-associated vasculitis. Ann Rheum Dis.

[9] Talar-Williams C, Hijazi YM, Walther MM (1996). Cyclophosphamide-induced cystitis and bladder cancer in patients with Wegener granulomatosis. Ann Intern Med.

[10] Knight A, Askling J, Ekbom A (2002). Cancer incidence in a population-based cohort of patients with Wegener’s granulomatosis. Int J cancer.

[11] Faurschou M, Sorensen IJ, Mellemkjaer L (2008). Malignancies in Wegener’s granulomatosis: incidence and relation to cyclophosphamide therapy in a cohort of 293 patients. J Rheumatol.

[12] Faurschou M, Mellemkjaer L, Voss A (2015). Prolonged risk of specific malignancies following cyclophosphamide therapy among patients with granulomatosis with polyangiitis. Rheumatology (Oxford).

[13] Zycinska K, Kostrzewa-Janicka J, Nitsch-Osuch A, Wardyn K (2013). Cancer incidence in pulmonary vasculitis. Adv Exp Med Biol.

[14] Westman KW, Bygren PG, Olsson H (1998). Relapse rate, renal survival, and cancer morbidity in patients with Wegener’s granulomatosis or microscopic polyangiitis with renal involvement. J Am Soc Nephrol.

[15] Knight A, Askling J, Granath F (2004). Urinary bladder cancer in Wegener’s granulomatosis: risks and relation to cyclophosphamide. Ann Rheum Dis.

[16] Rahmattulla C, Berden AE, Wakker S-C (2015). Incidence of malignancies in patients with antineutrophil cytoplasmic antibody-associated vasculitis diagnosed between 1991 and 2013. Arthritis Rheumatol (Hoboken, NJ).

[17] Pankhurst T, Savage COS, Gordon C, Harper L (2004). Malignancy is increased in ANCA-associated vasculitis. Rheumatology (Oxford).

[18] Shang W, Ning Y, Xu X (2015). Incidence of cancer in ANCA-associated vasculitis: a meta-analysis of observational studies. PLoS ONE.

[19] Mahr A, Heijl C, Le Guenno G, Faurschou M (2013). ANCA-associated vasculitis and malignancy: current evidence for cause and consequence relationships. Best Pract Res Clin Rheumatol.

[20] Jayne D, Rasmussen N, Andrassy K (2003). A randomized trial of maintenance therapy for vasculitis associated with antineutrophil cytoplasmic autoantibodies. N Engl J Med.

[21] Heijl C, Harper L, Flossmann O (2011). Incidence of malignancy in patients treated for antineutrophil cytoplasm antibody-associated vasculitis: follow-up data from European Vasculitis Study Group clinical trials. Ann Rheum Dis.

[22] Sastre López A, Íñigo Vanrell V, Gascó Company JM (2008). Granulomatosis de Wegener y cáncer. Nefrologia.

[23] Toriyama M, Tagaya E, Yamamoto T (2018). Lung cancer development in the patient with granulomatosis with polyangiitis during long term treatment with cyclophosphamide: first documented case. Respirol Case Rep.

[24] Doberstein T, Swick BL, Singh N (2017). Skin nodule reveals lung cancer in a patient with granulomatosis with polyangiitis. Clin Case Rep.

[25] Xie C, Stoddart C, Bewes J (2019). A hidden lung cancer in a patient with granulomatosis with polyangiitis. BJR Case Rep.

[26] Chemouny JM, Pagnoux C, Caudwell V (2014). ANCA-associated diseases and lung carcinomas: a five-case series. Clin Nephrol.

[27] Gafoor K, Patel S, Girvin F (2018). Cavitary lung diseases: a clinical-radiologic algorithmic approach. Chest.

[28] Fain O, Hamidou M, Cacoub P (2007). Vasculitides associated with malignancies: analysis of sixty patients. Arthritis Rheum.

[29] Morisako T, Tsuchida F, Nakamura H (2006). A case of squamous cell carcinoma of the lung with a high titer of proteinase 3 antineutrophil cytoplasmic antibody. Nihon Kokyuki Gakkai Zasshi.

[30] Okauchi S, Tamura T, Kagohashi K (2016). Elevated serum levels of two anti-neutrophil cytoplasmic antibodies in a lung cancer patient: a case report. Biomed Rep.

[31] Tsuchiya K, Karayama M, Sato T (2019). Simultaneous occurrence of sarcoidosis and anti-neutrophil cytoplasmic antibody-associated vasculitis in a patient with lung cancer. Intern Med.

[32] Edgar JD, Rooney DP, McNamee P, McNeill TA (1993). An association between ANCA positive renal disease and malignancy. Clin Nephrol.

[33] Tatsis E, Reinhold-Keller E, Steindorf K (1999). Wegener’s granulomatosis associated with renal cell carcinoma. Arthritis Rheum.

[34] Sriskandarajah S, Bostad L, Myklebust TÅ (2017). Cancer in ANCA-associated glomerulonephritis: a registry-based cohort study. Int J Nephrol.

[35] Jardel S, Puéchal X, Le Quellec A (2018). Mortality in systemic necrotizing vasculitides: a retrospective analysis of the French Vasculitis Study Group registry. Autoimmun Rev.

[36] Klinaki E, Katsoulis M, La Vecchia C, Trichopoulou A (2018). Rheumatoid arthritis and cancer risk results from the Greek European prospective investigation into cancer and nutrition cohort. Eur J Cancer Prev.

[37] Song L, Wang Y, Zhang J (2018). The risks of cancer development in systemic lupus erythematosus (SLE) patients: a systematic review and meta-analysis. Arthritis Res Ther.

[38] Onishi A, Sugiyama D, Kumagai S, Morinobu A (2013). Cancer incidence in systemic sclerosis: meta-analysis of population-based cohort studies. Arthritis Rheum.

[39] Theander E, Henriksson G, Ljungberg O (2006). Lymphoma and other malignancies in primary Sjogren’s syndrome: a cohort study on cancer incidence and lymphoma predictors. Ann Rheum Dis.

[40] Hemminki K, Liu X, Ji J (2011). Subsequent COPD and lung cancer in patients with autoimmune disease. Eur Respir J.

[41] Yu K-H, Kuo C-F, Huang LH (2016). Cancer risk in patients with inflammatory systemic autoimmune rheumatic diseases. Medicine (Baltimore).

[42] Cappelli LC, Shah AA (2020). The relationships between cancer and autoimmune rheumatic diseases. Best Pract Res Clin Rheumatol.

[43] Ahlmann M, Hempel G (2016). The effect of cyclophosphamide on the immune system: implications for clinical cancer therapy. Cancer Chemother Pharmacol.

[44] Pugh D, Farrah TE, Gallacher PJ (2019). Cyclophosphamide-Induced Lung Injury. Kidney Int Rep.

[45] Kim R, Emi M, Tanabe K (2006). Cancer immunosuppression and autoimmune disease: beyond immunosuppressive networks for tumour immunity. Immunology.

[46] Franks AL, Slansky JE (2012). Multiple associations between a broad spectrum of autoimmune diseases, chronic inflammatory diseases and cancer. Anticancer Res.

[47] Korniluk A, Koper O, Kemona H, Dymicka-Piekarska V (2017). From inflammation to cancer. Ir J Med Sci.

[48] Rani L, Minz RW, Sharma A (2015). Predominance of PR3 specific immune response and skewed TH17 vs. T-regulatory milieu in active granulomatosis with polyangiitis. Cytokine.

[49] Nogueira E, Hamour S, Sawant D (2010). Serum IL-17 and IL-23 levels and autoantigen-specific Th17 cells are elevated in patients with ANCA-associated vasculitis. Nephrol Dial Transplant Off Publ Eur Dial Transpl Assoc Eur Ren Assoc.

[50] Cam C, Karagoz B, Muftuoglu T (2016). The inflammatory cytokine interleukin-23 is elevated in lung cancer, particularly small cell type. Contemp Oncol (Poznan, Poland).

[51] Marrugal Á, Ojeda L, Paz-Ares L (2016). Proteomic-based approaches for the study of cytokines in lung cancer. Dis Markers.

[52] Kallberg H, Padyukov L, Plenge RM (2007). Gene-gene and gene-environment interactions involving HLA-DRB1, PTPN22, and smoking in two subsets of rheumatoid arthritis. Am J Hum Genet.

[53] Yamaguchi M, Ando M, Katsuno T (2018). Smoking is a risk factor for relapse of antimyeloperoxidase antibodies-associated vasculitis. J Clin Rheumatol Pract Rep Rheum Musculoskelet Dis.

[54] Costenbader KH, Karlson EW (2006). Cigarette smoking and autoimmune disease: what can we learn from epidemiology?. Lupus.

[55] Khabbazi A, Alinejati B, Hajialilo M (2019). Cigarette smoking and risk of primary systemic vasculitis: a propensity score matching analysis. Sarcoidosis Vasc Diffus Lung Dis.

[56] Alberg AJ, Brock MV, Ford JG (2013). Epidemiology of lung cancer: Diagnosis and management of lung cancer, 3rd ed: American college of chest physicians evidence-based clinical practice guidelines. Chest.

[57] Jassem E, Szymanowska A, Siemińska A, Jassem J (2009). Smoking and lung cancer. Pneumonol Alergol Pol.

[58] Thet Z, Lam AK, Ranganathan D (2020). Cancer risks along the disease trajectory in antineutrophil cytoplasmic antibody associated vasculitis. Clin Rheumatol.

